# 1,5-Bis(2-meth­oxy­benzyl­idene)thio­carbonohydrazide methanol monosolvate

**DOI:** 10.1107/S1600536813016954

**Published:** 2013-06-22

**Authors:** Jianfeng Yu, Shiming Tang, Jingbin Zeng, Zifeng Yan

**Affiliations:** a Department of Chemistry, College of Science, China University of Petroleum, Qingdao 266555, People’s Republic of China; b Sate Key Laboratory of Heavy Oil Processing, China University of Petroleum, Qingdao 266555, People’s Republic of China

## Abstract

The title compound, C_17_H_18_N_4_O_2_S·CH_3_OH, was synthesized by the condensation reaction of *o*-meth­oxy­benzaldehyde with thio­carbohydrazide in methanol. The two benzene rings are inclined each to other at 31.7 (1)°. Inter­molecular N—H⋯O and bifurcated O—H⋯N(S) hydrogen bonds link two thio­carbonohydrazide and two solvent mol­ecules into a centrosymmetric unit. These units, related by translation along the *b* axis, are further aggregated into columns through N—H⋯S hydrogen bonds.

## Related literature

For biological activities of thio­carbohydrazides, see: Liang (2003 ▶); Bacchi *et al.* (2005 ▶). For the crystal structures of related compounds, see: Fang *et al.* (2006 ▶); Feng *et al.* (2011 ▶); Zhao (2011 ▶).
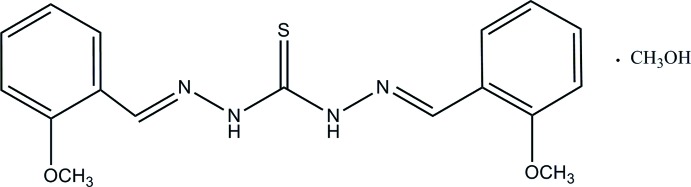


## Experimental

### 

#### Crystal data

C_17_H_18_N_4_O_2_S·CH_4_O
*M* *_r_* = 374.46
Triclinic, 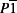

*a* = 7.7223 (15) Å
*b* = 10.232 (2) Å
*c* = 12.648 (3) Å
α = 85.938 (3)°
β = 80.796 (3)°
γ = 79.550 (3)°
*V* = 969.3 (3) Å^3^
*Z* = 2
Mo *K*α radiation
μ = 0.19 mm^−1^
*T* = 296 K
0.25 × 0.21 × 0.18 mm

#### Data collection

Bruker SMART APEX CCD area-detector diffractometer
Absorption correction: multi-scan (*SADABS*; Bruker, 2007 ▶) *T*_min_ = 0.954, *T*_max_ = 0.966
4769 measured reflections
3324 independent reflections
2766 reflections with *I* > 2σ(*I*)
*R*_int_ = 0.024

#### Refinement

*R*[*F* ^2^ > 2σ(*F* ^2^)] = 0.049
*wR*(*F* ^2^) = 0.171
*S* = 1.01
3324 reflections
240 parameters
H-atom parameters constrained
Δρ_max_ = 0.20 e Å^−3^
Δρ_min_ = −0.21 e Å^−3^

### 

Data collection: *SMART* (Bruker, 2007 ▶); cell refinement: *SAINT* (Bruker, 2007 ▶); data reduction: *SAINT*; program(s) used to solve structure: *SHELXS97* (Sheldrick, 2008 ▶); program(s) used to refine structure: *SHELXL97* (Sheldrick, 2008 ▶); molecular graphics: *SHELXTL* (Sheldrick, 2008 ▶); software used to prepare material for publication: *SHELXTL*.

## Supplementary Material

Crystal structure: contains datablock(s) I, global. DOI: 10.1107/S1600536813016954/cv5415sup1.cif

Structure factors: contains datablock(s) I. DOI: 10.1107/S1600536813016954/cv5415Isup2.hkl

Supplementary material file. DOI: 10.1107/S1600536813016954/cv5415Isup3.cml

Additional supplementary materials:  crystallographic information; 3D view; checkCIF report

## Figures and Tables

**Table 1:** Hydrogen-bond geometry (Å, °)

*D*—H⋯*A*	*D*—H	H⋯*A*	*D*⋯*A*	*D*—H⋯*A*
O3—H3⋯S1^i^	0.82	2.80	3.534 (2)	150
O3—H3⋯N4^i^	0.82	2.36	3.028 (3)	139
N3—H3*A*⋯O3	0.86	2.38	3.126 (3)	145
N1—H1⋯S1^ii^	0.86	2.57	3.4184 (19)	169

Symmetry codes: (i) 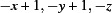; (ii) 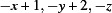.

**Fig. 1.**
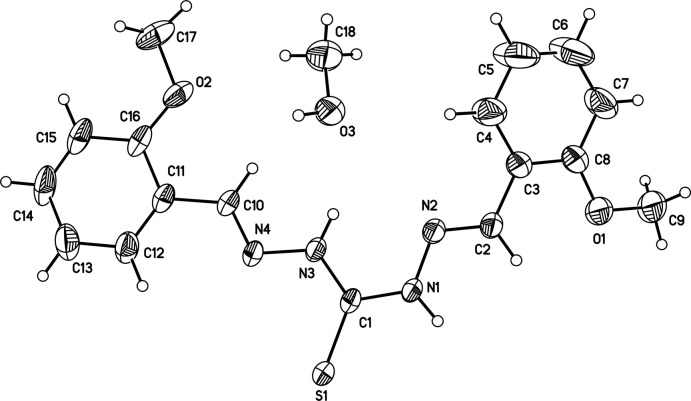
The molecular structure of (I) showing the atomic numbering and 30% probability displacement ellipsoids.

## supplementary crystallographic information

### Comment

Schiff bases of thiocarbohydrazide are impotant organic intermediates owing to their biological activities (Liang, 2003; Bacchi *et al.*, 2005). In a continuation of structural study of Schiff bases of thiocarbohydrazide (Fang *et al.*, 2006; Feng *et al.*, 2011; Zhao, 2011), we present here the title compound (I).

In (I) (Fig. 1), the bond lengths and angles are normal and correspond to those observed in 1,5-bis[(1E)-(2-methoxyphenyl)methylene]- thiocarbonohydrazide ethanol solvate (Fang, *et al.*, 2006), *N*'',*N*'''-bis(1-phenylethylidene)thiocarbonohydrazide (Feng *et al.*, 2011) and *N*'',*N*'''-bis(4-methoxybenzylidene)thiocarbonohydrazide methanol solvate (Zhao, 2011).

In the crystal, the benzene ring C3—C8 and the benzene ring C11—C16 are inclined each to other at 31.7 (1)°. In the crystal, intermolecular N—H···O and bifurcated O—H···N(S) hydrogen bonds (Table 1) link two *M* and two solvent molecules into centrosymmetric unit. These units related by translation along the *b* axis are futher aggregated into columns through the N—H···S hydrogen bonds (Table 1).

### Experimental

A 50 ml flask was charged with a magnetic stir bar, *o*-methoxybenzaldehyde (1 mmol), thiocarbohydrazide (0.5 mmol) in 20 ml me thanol.After stirring 3 h at 373 K, the resulting mixture was cooled to room temperature, and recrystalized from methanol, and afforded the title compound as a crystalline solid.

### Refinement

All H atoms were placed in geometrically idealized positions (C—H 0.93–0.96 Å, N—H 0.86 Å, O—H 0.82 Å) and treated as riding on their parent atoms, with *U*_iso_(H) = 1.2–1.5*U*_eq_(C,*O*, N).

### Crystal data

**Table:**

C_17_H_18_N_4_O_2_S·CH_4_O	*Z* = 2
*M**_r_* = 374.46	*F*(000) = 396
Triclinic, *P*1	*D*_x_ = 1.283 Mg m^−^^3^
*a* = 7.7223 (15) Å	Mo *K*α radiation, λ = 0.71073 Å
*b* = 10.232 (2) Å	Cell parameters from 2955 reflections
*c* = 12.648 (3) Å	θ = 3.0–28.1°
α = 85.938 (3)°	µ = 0.19 mm^−^^1^
β = 80.796 (3)°	*T* = 296 K
γ = 79.550 (3)°	Block, colourless
*V* = 969.3 (3) Å^3^	0.25 × 0.21 × 0.18 mm

### Data collection

**Table:**

Bruker SMART APEX CCD area-detector diffractometer	3324 independent reflections
Radiation source: fine-focus sealed tube	2766 reflections with *I* > 2σ(*I*)
Graphite monochromator	*R*_int_ = 0.024
phi and ω scans	θ_max_ = 25.0°, θ_min_ = 1.6°
Absorption correction: multi-scan (*SADABS*; Bruker, 2007)	*h* = −8→9
*T*_min_ = 0.954, *T*_max_ = 0.966	*k* = −9→12
4769 measured reflections	*l* = −15→13

### Refinement

**Table:**

Refinement on *F*^2^	Secondary atom site location: difference Fourier map
Least-squares matrix: full	Hydrogen site location: inferred from neighbouring sites
*R*[*F*^2^ > 2σ(*F*^2^)] = 0.049	H-atom parameters constrained
*wR*(*F*^2^) = 0.171	*w* = 1/[σ^2^(*F*_o_^2^) + (0.1*P*)^2^ + 0.3851*P*] where *P* = (*F*_o_^2^ + 2*F*_c_^2^)/3
*S* = 1.01	(Δ/σ)_max_ = 0.010
3324 reflections	Δρ_max_ = 0.20 e Å^−^^3^
240 parameters	Δρ_min_ = −0.21 e Å^−^^3^
0 restraints	Extinction correction: *SHELXL97* (Sheldrick, 2008), Fc^*^=kFc[1+0.001xFc^2^λ^3^/sin(2θ)]^-1/4^
Primary atom site location: structure-invariant direct methods	Extinction coefficient: 0.038 (7)

### Special details

**Table:**

Geometry. All e.s.d.'s (except the e.s.d. in the dihedral angle between two l.s. planes) are estimated using the full covariance matrix. The cell e.s.d.'s are taken into account individually in the estimation of e.s.d.'s in distances, angles and torsion angles; correlations between e.s.d.'s in cell parameters are only used when they are defined by crystal symmetry. An approximate (isotropic) treatment of cell e.s.d.'s is used for estimating e.s.d.'s involving l.s. planes.
Refinement. Refinement of *F*^2^ against ALL reflections. The weighted *R*-factor *wR* and goodness of fit *S* are based on *F*^2^, conventional *R*-factors *R* are based on *F*, with *F* set to zero for negative *F*^2^. The threshold expression of *F*^2^ > σ(*F*^2^) is used only for calculating *R*-factors(gt) *etc*. and is not relevant to the choice of reflections for refinement. *R*-factors based on *F*^2^ are statistically about twice as large as those based on *F*, and *R*- factors based on ALL data will be even larger.

### Fractional atomic coordinates and isotropic or equivalent isotropic displacement parameters (Å^2^)

**Table:**

	*x*	*y*	*z*	*U*_iso_*/*U*_eq_	
N1	0.4120 (3)	0.85311 (18)	0.10378 (15)	0.0503 (5)	
H1	0.4497	0.9278	0.0942	0.060*	
N2	0.3689 (3)	0.80149 (19)	0.20555 (15)	0.0524 (5)	
N3	0.3445 (2)	0.66888 (17)	0.04305 (15)	0.0476 (5)	
H3A	0.3322	0.6395	0.1087	0.057*	
N4	0.3114 (2)	0.59365 (18)	−0.03557 (15)	0.0480 (5)	
O1	0.2688 (4)	1.0468 (2)	0.44226 (17)	0.1014 (9)	
O2	0.1142 (3)	0.27193 (17)	0.08315 (18)	0.0739 (6)	
S1	0.43309 (9)	0.85466 (6)	−0.10545 (5)	0.0567 (3)	
C1	0.3953 (3)	0.7867 (2)	0.01844 (18)	0.0438 (5)	
C2	0.3709 (3)	0.8750 (3)	0.28184 (19)	0.0574 (6)	
H2	0.3972	0.9601	0.2666	0.069*	
C3	0.3324 (4)	0.8276 (3)	0.3932 (2)	0.0644 (7)	
C4	0.3466 (6)	0.6932 (3)	0.4199 (3)	0.0963 (11)	
H4	0.3800	0.6319	0.3662	0.116*	
C5	0.3117 (8)	0.6491 (4)	0.5254 (3)	0.144 (2)	
H5	0.3207	0.5585	0.5428	0.173*	
C6	0.2634 (8)	0.7397 (5)	0.6049 (3)	0.137 (2)	
H6	0.2389	0.7098	0.6760	0.164*	
C7	0.2510 (6)	0.8722 (4)	0.5809 (3)	0.1038 (12)	
H7	0.2214	0.9324	0.6354	0.125*	
C8	0.2826 (4)	0.9174 (3)	0.4751 (2)	0.0720 (7)	
C9	0.2107 (7)	1.1424 (4)	0.5220 (3)	0.1161 (15)	
H9A	0.0990	1.1274	0.5620	0.174*	
H9B	0.1962	1.2301	0.4885	0.174*	
H9C	0.2977	1.1346	0.5695	0.174*	
C10	0.2413 (3)	0.4934 (2)	0.0006 (2)	0.0487 (5)	
H10	0.2192	0.4771	0.0744	0.058*	
C11	0.1943 (3)	0.4031 (2)	−0.0700 (2)	0.0543 (6)	
C12	0.2091 (4)	0.4269 (3)	−0.1782 (2)	0.0731 (8)	
H12	0.2533	0.5019	−0.2087	0.088*	
C13	0.1593 (5)	0.3415 (4)	−0.2435 (3)	0.0937 (11)	
H13	0.1698	0.3585	−0.3173	0.112*	
C14	0.0940 (4)	0.2309 (4)	−0.1973 (3)	0.0958 (12)	
H14	0.0581	0.1741	−0.2404	0.115*	
C15	0.0809 (4)	0.2032 (3)	−0.0901 (3)	0.0804 (9)	
H15	0.0392	0.1268	−0.0607	0.097*	
C16	0.1297 (3)	0.2888 (2)	−0.0246 (3)	0.0600 (7)	
C17	0.0616 (5)	0.1520 (3)	0.1332 (4)	0.0952 (11)	
H17A	−0.0618	0.1534	0.1290	0.143*	
H17B	0.0787	0.1458	0.2070	0.143*	
H17C	0.1326	0.0766	0.0970	0.143*	
O3	0.3985 (3)	0.4521 (2)	0.22744 (16)	0.0803 (6)	
H3	0.4610	0.4009	0.1842	0.121*	
C18	0.2837 (6)	0.3838 (4)	0.2970 (3)	0.1132 (14)	
H18A	0.3517	0.3184	0.3393	0.170*	
H18B	0.2173	0.3406	0.2563	0.170*	
H18C	0.2031	0.4454	0.3434	0.170*	

### Atomic displacement parameters (Å^2^)

**Table:**

	*U*^11^	*U*^22^	*U*^33^	*U*^12^	*U*^13^	*U*^23^
N1	0.0627 (11)	0.0401 (10)	0.0515 (11)	−0.0201 (8)	−0.0034 (8)	−0.0085 (8)
N2	0.0588 (11)	0.0468 (10)	0.0529 (11)	−0.0149 (9)	−0.0028 (8)	−0.0086 (9)
N3	0.0554 (10)	0.0389 (9)	0.0514 (10)	−0.0155 (8)	−0.0049 (8)	−0.0098 (8)
N4	0.0496 (10)	0.0398 (9)	0.0569 (11)	−0.0113 (8)	−0.0059 (8)	−0.0129 (8)
O1	0.172 (2)	0.0723 (14)	0.0597 (12)	−0.0446 (15)	0.0175 (13)	−0.0220 (10)
O2	0.0782 (12)	0.0486 (10)	0.1034 (16)	−0.0266 (9)	−0.0268 (11)	0.0098 (10)
S1	0.0740 (4)	0.0492 (4)	0.0532 (4)	−0.0275 (3)	−0.0076 (3)	−0.0062 (3)
C1	0.0403 (10)	0.0361 (10)	0.0555 (12)	−0.0073 (8)	−0.0049 (9)	−0.0098 (9)
C2	0.0680 (14)	0.0541 (13)	0.0526 (13)	−0.0203 (11)	−0.0022 (11)	−0.0106 (11)
C3	0.0760 (16)	0.0634 (16)	0.0527 (14)	−0.0142 (13)	−0.0024 (12)	−0.0053 (12)
C4	0.140 (3)	0.0634 (18)	0.0710 (19)	−0.0009 (19)	0.0064 (19)	−0.0020 (15)
C5	0.235 (6)	0.077 (2)	0.085 (3)	0.015 (3)	0.022 (3)	0.022 (2)
C6	0.212 (5)	0.102 (3)	0.066 (2)	0.021 (3)	0.007 (3)	0.017 (2)
C7	0.146 (3)	0.101 (3)	0.0549 (17)	−0.007 (2)	−0.0002 (19)	−0.0093 (17)
C8	0.0876 (19)	0.0748 (18)	0.0527 (14)	−0.0158 (15)	−0.0034 (13)	−0.0082 (13)
C9	0.188 (4)	0.087 (2)	0.074 (2)	−0.047 (3)	0.019 (2)	−0.0335 (19)
C10	0.0453 (11)	0.0381 (11)	0.0653 (14)	−0.0105 (9)	−0.0103 (10)	−0.0080 (10)
C11	0.0439 (11)	0.0452 (12)	0.0758 (16)	−0.0115 (9)	−0.0040 (10)	−0.0185 (11)
C12	0.0740 (17)	0.0731 (18)	0.0786 (19)	−0.0300 (14)	−0.0008 (14)	−0.0262 (15)
C13	0.098 (2)	0.109 (3)	0.083 (2)	−0.042 (2)	0.0038 (17)	−0.044 (2)
C14	0.085 (2)	0.095 (2)	0.118 (3)	−0.0436 (19)	0.0095 (19)	−0.062 (2)
C15	0.0646 (16)	0.0599 (16)	0.122 (3)	−0.0255 (13)	0.0028 (16)	−0.0369 (17)
C16	0.0412 (11)	0.0383 (12)	0.102 (2)	−0.0056 (9)	−0.0084 (12)	−0.0193 (12)
C17	0.082 (2)	0.0633 (18)	0.148 (3)	−0.0364 (16)	−0.026 (2)	0.026 (2)
O3	0.1011 (15)	0.0707 (13)	0.0658 (12)	−0.0183 (11)	0.0032 (10)	−0.0064 (10)
C18	0.152 (4)	0.103 (3)	0.084 (2)	−0.050 (3)	0.014 (2)	0.000 (2)

### Geometric parameters (Å, º)

**Table:**

N1—C1	1.351 (3)	C7—H7	0.9300
N1—N2	1.370 (3)	C9—H9A	0.9600
N1—H1	0.8600	C9—H9B	0.9600
N2—C2	1.268 (3)	C9—H9C	0.9600
N3—C1	1.335 (3)	C10—C11	1.458 (3)
N3—N4	1.381 (2)	C10—H10	0.9300
N3—H3A	0.8600	C11—C12	1.363 (4)
N4—C10	1.269 (3)	C11—C16	1.404 (3)
O1—C8	1.350 (4)	C12—C13	1.386 (4)
O1—C9	1.419 (4)	C12—H12	0.9300
O2—C16	1.350 (4)	C13—C14	1.377 (5)
O2—C17	1.435 (3)	C13—H13	0.9300
S1—C1	1.672 (2)	C14—C15	1.356 (5)
C2—C3	1.459 (4)	C14—H14	0.9300
C2—H2	0.9300	C15—C16	1.385 (4)
C3—C4	1.382 (4)	C15—H15	0.9300
C3—C8	1.395 (4)	C17—H17A	0.9600
C4—C5	1.379 (5)	C17—H17B	0.9600
C4—H4	0.9300	C17—H17C	0.9600
C5—C6	1.378 (6)	O3—C18	1.391 (4)
C5—H5	0.9300	O3—H3	0.8200
C6—C7	1.358 (6)	C18—H18A	0.9600
C6—H6	0.9300	C18—H18B	0.9600
C7—C8	1.383 (4)	C18—H18C	0.9600
			
C1—N1—N2	119.98 (18)	O1—C9—H9C	109.5
C1—N1—H1	120.0	H9A—C9—H9C	109.5
N2—N1—H1	120.0	H9B—C9—H9C	109.5
C2—N2—N1	116.55 (19)	N4—C10—C11	122.0 (2)
C1—N3—N4	120.89 (19)	N4—C10—H10	119.0
C1—N3—H3A	119.6	C11—C10—H10	119.0
N4—N3—H3A	119.6	C12—C11—C16	119.1 (2)
C10—N4—N3	113.93 (19)	C12—C11—C10	122.2 (2)
C8—O1—C9	117.2 (3)	C16—C11—C10	118.7 (2)
C16—O2—C17	118.8 (3)	C11—C12—C13	121.2 (3)
N3—C1—N1	114.5 (2)	C11—C12—H12	119.4
N3—C1—S1	125.35 (17)	C13—C12—H12	119.4
N1—C1—S1	120.10 (16)	C14—C13—C12	118.8 (4)
N2—C2—C3	120.8 (2)	C14—C13—H13	120.6
N2—C2—H2	119.6	C12—C13—H13	120.6
C3—C2—H2	119.6	C15—C14—C13	121.4 (3)
C4—C3—C8	118.6 (3)	C15—C14—H14	119.3
C4—C3—C2	120.9 (3)	C13—C14—H14	119.3
C8—C3—C2	120.5 (2)	C14—C15—C16	119.9 (3)
C5—C4—C3	120.5 (3)	C14—C15—H15	120.0
C5—C4—H4	119.7	C16—C15—H15	120.0
C3—C4—H4	119.7	O2—C16—C15	123.9 (3)
C6—C5—C4	119.7 (4)	O2—C16—C11	116.5 (2)
C6—C5—H5	120.1	C15—C16—C11	119.5 (3)
C4—C5—H5	120.1	O2—C17—H17A	109.5
C7—C6—C5	120.8 (4)	O2—C17—H17B	109.5
C7—C6—H6	119.6	H17A—C17—H17B	109.5
C5—C6—H6	119.6	O2—C17—H17C	109.5
C6—C7—C8	119.8 (3)	H17A—C17—H17C	109.5
C6—C7—H7	120.1	H17B—C17—H17C	109.5
C8—C7—H7	120.1	C18—O3—H3	109.5
O1—C8—C7	124.6 (3)	O3—C18—H18A	109.5
O1—C8—C3	115.0 (2)	O3—C18—H18B	109.5
C7—C8—C3	120.4 (3)	H18A—C18—H18B	109.5
O1—C9—H9A	109.5	O3—C18—H18C	109.5
O1—C9—H9B	109.5	H18A—C18—H18C	109.5
H9A—C9—H9B	109.5	H18B—C18—H18C	109.5

### Hydrogen-bond geometry (Å, º)

**Table:**

*D*—H···*A*	*D*—H	H···*A*	*D*···*A*	*D*—H···*A*
O3—H3···S1^i^	0.82	2.80	3.534 (2)	150
O3—H3···N4^i^	0.82	2.36	3.028 (3)	139
N3—H3*A*···O3	0.86	2.38	3.126 (3)	145
N1—H1···S1^ii^	0.86	2.57	3.4184 (19)	169
N3—H3*A*···N2	0.86	2.21	2.591 (3)	106

Symmetry codes: (i) −*x*+1, −*y*+1, −*z*; (ii) −*x*+1, −*y*+2, −*z*.
